# HIF2α induces cardiomyogenesis via Wnt/β-catenin signaling in mouse embryonic stem cells

**DOI:** 10.1186/s12967-015-0447-7

**Published:** 2015-03-14

**Authors:** Xiaotian Sun, Liewen Pang, Meng Shi, Jiechun Huang, Yiqing Wang

**Affiliations:** Department of Cardiothoracic surgery, Huashan Hospital of Fudan University, 12th Wulumuqi Road, Shanghai, 200040 PR China

**Keywords:** Embryonic stem cells, Cardiomyogenesis, HIF2α, β-catenin

## Abstract

**Background:**

Embryonic stem cells (ESCs) are pluripotent stem cells and can differentiate into cardiomyocytes when cultured in appropriate conditions. The function of hypoxia-inducible factors (HIFs) has been identified in directing the formation of cardiac lineages. The purpose of this study was to investigate the ability of HIF2α to induce differentiation of ESCs into cardiomyocytes and to explore the potential underlying molecular mechanisms.

**Methods:**

Cardiac differentiation from mouse ESCs was analyzed using the “hanging drop” method, and success was determined by assaying the numbers of beating embryoid bodies and the expression level of cardiac markers. The expression of *HIF2α* was then manipulated during cardiac differentiation with piggyBac transposon and the lentivirus system. The underlying mechanism was finally examined via administering selective inhibitors of the Wnt/β-catenin signaling pathway.

**Results:**

Overexpressing *HIF2α* can significantly drive mouse ESCs to form cardiomyocytes. Contrarily, knockdown of *HIF2α* inhibits the emergence of cardiac cells. In addition, the cardiomyogenesis-promoting effect of HIF2α occurred by increasing the protein level of β-catenin, an effector that contributes to cardiac differentiation at an early stage of ESC differentiation.

**Conclusion:**

*HIF2α* has a cardiomyogenesis-promoting effect in ESCs via enhancing the activation of the Wnt/β-catenin signaling pathway. Our results may be beneficial for generating and applying cardiomyocytes from ESCs safely and effectively in the future.

**Electronic supplementary material:**

The online version of this article (doi:10.1186/s12967-015-0447-7) contains supplementary material, which is available to authorized users.

## Background

Cardiomyogenic development is a multistep process involving a cascade of signaling events and multiple transcription factors, which operate in a spatial and temporal fashion to specify the fate of cardiac cells [1]. Unfortunately, our current understanding of cardiomyogenesis is limited due to the difficulty of studying cardiac specification effectively in vivo. In vitro manipulation of embryonic stem cells (ESCs) to form embryoid bodies (EBs) that consistently give rise to beating cardiomyocytes has become a useful model for exploring the structural and functional properties of cardiomyogenesis [2]. ESCs are derived from the inner cell mass of pre-implantation blastocysts and can be maintained in culture indefinitely while retaining the capacity to generate nearly any type of cell in the body [3,4]. These beating cells have become an attractive candidate cell source to obtain enough cardiomyocytes for cardiac repair, such as after myocardial infarction, which is characterized by the loss of functional cardiomyocytes [5]. However, the molecular mechanisms governing differentiation of ESCs into cardiomyocytes are still poorly understood.

Cardiac fate decisions are influenced by many parameters, such as FGF [6], Notch [7], Wnt [8] signaling pathways, GATA transcription factors [9] and hypoxia conditions [10]. Actually, hypoxia is involved the natural progression of organogenesis in the early development of mammals and is one of the most critical elements for the formation of the heart [11]. Consistently, low oxygen tension in vitro has been shown to enhance cardiac differentiation from ESCs [12,13]. The pivotal regulators of cellular responses to oxygen deprivation are hypoxia-inducible factors (HIFs) [14], which are heterodimeric transcription factors including HIF1α, HIF2α and HIF3α [14]. Of interest, HIF1α is more similar to HIF2α than HIF3α in functional domains, expression patterns and functions. Both HIF1α and HIF2α proteins contain the basic-helix-loop-helix Per-ARNT-SIM domain (bHLH-PAS), which is critical for gene binding. In addition, both are widely expressed in various tissues. However, *HIF3α* is normally expressed only in highly avascular tissues, such as the cornea. Additionally, they activate gene transcription while HIF3α inhibits the HIF1α- or HIF2α-mediated hypoxia responses [14]. A previous study demonstrated that HIF1α is essential for proper cardiac differentiation because *HIF1α* deficiency leads to abnormal cardiac looping in mice due to defective ventricle formation caused by reduced expression of myocyte factors [11]. Similarly, cultured ESCs in vitro without HIF1α expression rarely form beating embryoid bodies (EBs) [15], while overexpression of *HIF1α* can promote cardiac differentiation in mouse ECS-derived EBs [16, 17]. Notably, both HIF1α and HIF2α protein complexes are expressed in cardiac tissue [18]. However, little is known about the role of HIF2α in cardiac differentiation.

In this study, we investigated the role of HIF2α in cardiac differentiation using gain- and loss-of-function methods in mouse ESCs, and explored the possible intracellular signaling pathways by which HIF2α activates this process. Our study might provide expanded insight to create an effective strategy for promoting differentiation of ESCs cells into cardiomyocytes.

## Methods

### Mouse ESC culture

46C ESCs, kindly provided by Dr. Smith A (University of Cambridge), were cultured on 0.1% gelatin-coated dishes at 37°C in 5% CO_2_. The medium for routine maintenance was GMEM (Sigma, G5414) supplemented with 10% FCS (HyClone), 1% MEM nonessential amino acids (Invitrogen), 2 mM GlutaMax (Invitrogen), 0.1 mM β-mercaptoethanol (Invitrogen) and 100 units/ml LIF (Millipore). Cells were digested by 0.25% trypsin (Invitrogen) and passaged when confluence reached approximately 70%.

### Cardiac differentiation of ESCs

ESCs were differentiated into beating cardiomyocytes in vitro by the “hanging drop” method as described previously [19]. Briefly, the modified steps included withdrawal of LIF and cultivation of 1,000 cells in 30 μL hanging drops to produce EBs for two days. After two days, the EBs were seeded onto gelatin-coated 48-well plates. The medium was renewed every two days. Over the next two weeks, the beating rates of these EBs were compared according to need.

### Plasmid construction and transfection

For RNA interference in ESCs, short hairpin (shRNA) constructs for *HIF2α* were designed to target 21 base-pair gene specific regions and were then amplified into the pLKO.1-TRC (AgeI and EcoRI sites). The targeted sequences are as follows: *HIF2α* sh#1:GCTTCCTTCGGACACATAAGC; *HIF2α* sh#2: GGGACTTACTCAGGTAGAACT. pLKO.1-TRC-based lentiviral vectors were transfected into 293 T cells in combination with pMD2.G and psPAX2 plasmids. Virus-containing supernatant was collected after 48 hours and filtered through 0.45 μm filters (Millipore). ESCs were incubated in the virus supernatant for 48 hours. For gene overexpression, the coding region of *HIF2α* was cloned from mouse cDNA with Hot Start DNA Polymerase (Takara) and was inserted into the BglП and SalI sites of the PiggyBac transposon vectors. ESCs were transfected with 2 μg PiggyBac inserted with targets plus a 2 μg transposon vector using Lipofectamine 2000 (Invitrogen) according to the manufacturer’s instructions. The modified cells were screened by treatment with 2 μg/ml puromycin for about one week.

### RT-PCR and qRT-PCR

Total RNA was extracted with TRIzol (Invitrogen). cDNA was synthesized with 1 μg of total RNA using a PrimeScript 1st strand cDNA Synthesis Kit (Takara) according to the manufacturer’s instructions. QRT-PCR was performed with SYBR® Premix Ex Taq™ (Takara) in an ABI7500 Real-Time PCR machine (Applied Biosystems). Target gene expression was normalized to GAPDH expression. The primers that were used are listed in Additional file 1: Table S1.

### Western blotting

Cells were lysed in ice-cold RIPA cell buffer (Sigma) supplemented with protease inhibitors (Sigma). The proteins were separated with a 4–12% PAGE gel and electrotransferred onto a PVDF membrane. The membrane was probed with primary antibodies and subsequently detected by horseradish-peroxidase (HRP) conjugated antibodies (Santa Cruz). The primary antibodies were β-catenin (Santa Cruz), Flag antibody (Sigma), HIF1α (Santa Cruz) and HIF2α (Santa Cruz).

### Luciferase reporter assay

TOP-Flash or FOP-Flash constructs (Addgene) were co-transfected with a Renilla luciferase plasmid (Promega) into mESCs overexpressing PB empty vectors or PB-HIF2α plasmids. Cells were harvested and the relative luciferase activity of the lysate was measured with the Dual-Luciferase reporter assay system (Promega).

### Immunofluorescence staining

Cells were fixed in 4% paraformaldehyde for 20 minutes and blocked in blocking buffer (PBS containing 5% BSA and 0.2% Triton X-100). Cells were incubated in primary antibody solution at 4°C overnight, followed by Alexa Fluor 488 (Invitrogen, 1:1000) secondary antibodies for 1 h at 37°C. Nuclei were stained with Hoechst (Invitrogen, 1:10000). The primary antibodies were Gata4 (G4; Santa Cruz, 1:100), Myosin (MF-20; DSHB, 1:50) and Troponin T (1:1000, Abcam).

### FACS analysis

Differentiated EBs were trypsinized into single cells, fixed in 2% PFA and permeabilized with 0.2% Triton × 100. Cells were suspended in antibody solution containing anti-cTnT and α-SMA antibodies (1:200, Abcam). Cells were washed in PBS three times and then resuspended in FITC-conjugated secondary antibody solution (1:1000, Invitrogen). Cardiomyocytes were detected with a BECKMAN COULTER FACS.

### Cobalt chloride (CoCl2) treatment

To induce a hypoxic condition, 100 μM CoCl2 (Sigma) were supplemented into ESCs or EBs. For analyzing the protein levels of HIF1α or HIF2α, 46C ESCs were treated with or without 100 μM CoCl2 for 10 h. For inducing cardiac differentiation, ESCs-derived EBs were exposed to 100 μM CoCl2 during the whole process of differentiation, and the medium was changed every two days.

### Statistical analysis

All data are reported as the mean ± s.d. A Student’s *t* test was used to determine the significance of differences in comparisons. Values of p < 0.05 were considered as statistically significant.

## Results and discussion

### HIF2a has no effect on the self-renewal of ESCs

**Figure 1:**
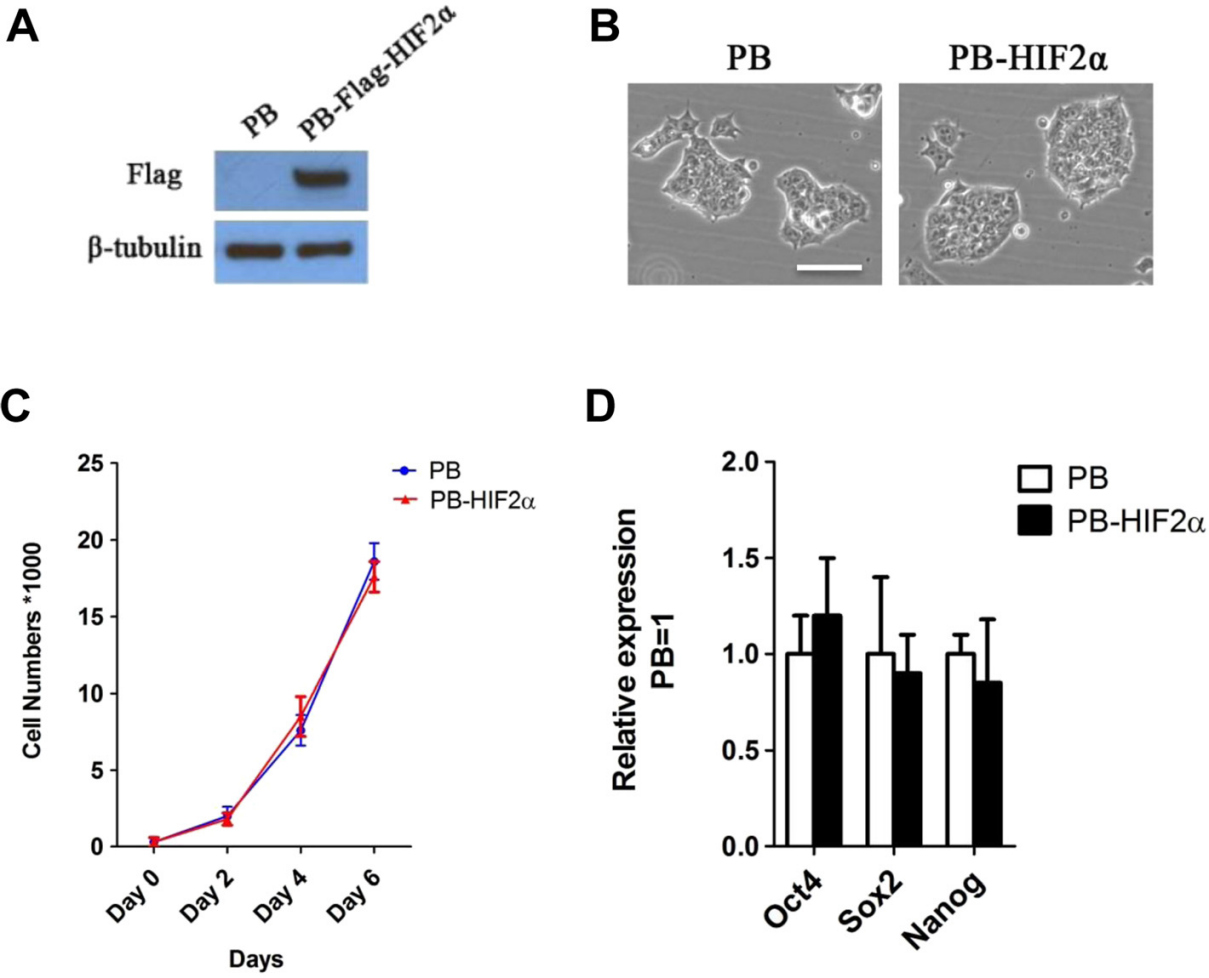
**The effect of HIF2α on the self-renewal of mouse ESCs. (A)** Western blot analysis of 46C mouse ESCs overexpressing empty vectors or Flag-tagged HIF2α. **(B)** Morphology of mouse ESCs transfected with empty or Flag-tagged *HIF2α* vectors and cultured in serum/LIF for five passages. Scale bar, 100 μm. **(C)** Cell growth assay. Relative cell proliferation was measured as a standard at Days 0, 2, 4 and 6. The original cell density was 3,000/well. Data represent the mean ± s.d. of three biological replicates. **(D)** Quantitative RT-PCR (qRT-PCR) analysis of *Oct4*, *Sox2* and *Nanog* in mouse ESCs transfected empty- or Flag-tagged HIF2α vectors. Data represent the mean ± s.d. of three biological replicates.

A previous report showed that HIF1α is sufficient to induce morphological changes reflective of the transition from ESCs to epiblast stem cells (EpiSCs) [20]. To investigate the function of HIF2α in the self-renewal of ESCs, mouse 46C ESCs were transfected with a piggyBac vector encoding *HIF2α* and analyzed by western blotting for expression of HIF2α (Figure 1A). There was no obvious distinguishing characteristic in the morphology of empty-vector control- and HIF2α-ESCs (Figure 1B). Additionally, HIF2α did not affect the proliferation of ESCs (Figure 1C) and the expression level of key pluripotency genes *Oct4*, *Sox2* and *Nanog* (Figure 1D). These results indicated that HIF2α does not affect the capacity for self-renewal of mouse ESCs.

### Expression pattern of HIF2a during cardiac differentiation

**Figure 2:**
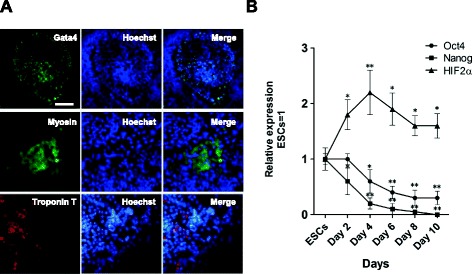
**Expression profile of** ***HIF2α*** **during cardiac differentiation. (A)** Immunostaining for cardiomyocyte markers Gata4, Myosin and Troponin T in mouse ESC-derived EBs at day 10. Scale bar, 100 μm. **(B)** qRT-PCR analysis of *Oct4*, *Nanog* and *HIF2α* in mouse ESCs and different days of ESCs-derived EBs. Data represent the mean ± s.d. of three biological replicates. *p < 0.05, **p < 0.01 vs ESCs.

To examine the expression profile of *HIF2α* during cardiac differentiation, we differentiated 46C ESCs into cardiomyocytes, which were immunopositive for cardiac markers Gata4, Myosin and Troponin T (Figure 2A), and then total RNA from ESCs and different stages of EBs (days 2, 4, 6, 8, and 10) were extracted and assessed by quantitative RT-PCR (qRT-PCR). qRT-PCR analysis showed that mRNA transcripts of pluripotency markers *Oct4* and *Nanog* were robust in undifferentiated mouse ESCs, but declined abruptly upon induction of EB formation (Figure 2B), while *HIF2α* was increased in differentiating EBs from day 2 onward and reached a peak at day 4 (Figure 2B), indicating that HIF2α may exert a role at an early stage of cardiac differentiation.

### HIF2α promotes cardiac differentiation of ESCs

**Figure 3:**
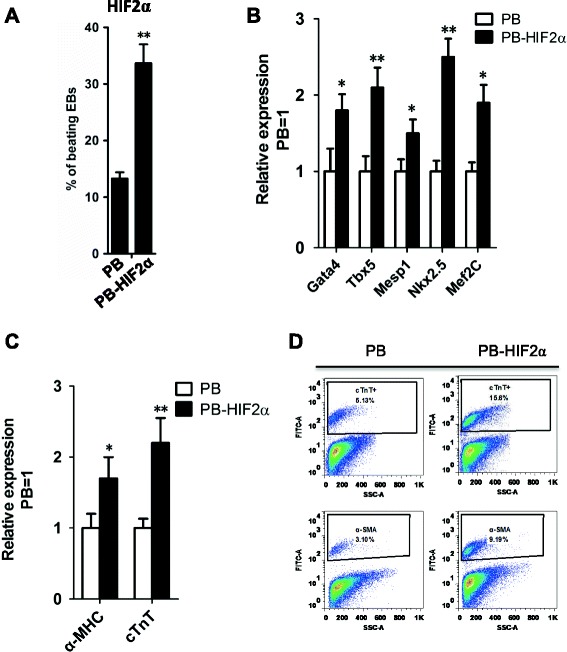
**HIF2α facilitated cardiac differentiation of ESCs. (A)** The percentages of beating EBs derived from PB and PB-*HIF2α* mouse ESCs. Data represent the mean ± s.d. of three biological replicates. **p < 0.01 vs PB. **(B)** qRT-PCR analysis of *Gata4*, *Tbx3*, *Mesp1*, *Nkx2.5* and *Mef2c* in PB and PB-HIF2α EBs at day 9. Data represent the mean ± s.d. of three biological replicates. *p < 0.05, **p < 0.01 vs PB. **(C)** qRT-PCR analysis of *α-MHC* and *cTnT* in PB- and PB-*HIF2α* EBs at day 15. Data represent the mean ± s.d. of three biological replicates. *p < 0.05, **p < 0.01 vs PB. **(D)** The differentiated cardiomyocytes from PB- and PB-*HIF2α* mouse ESCs were analyzed by FACS with cTnT and α-SMA staining.

Previous studies have shown that a hypoxic condition has strong influence during the development of the cardiovascular system, [17,21]. In line with this, hypoxia (4% oxygen tension) yields more cardiomyocytes than normoxic conditions in mouse and human ESCs [12,22]. As an important downstream target of hypoxia, we investigated the contribution of HIF2α in cardiomyogenesis. We differentiated empty-vector control- and HIF2α-ESCs into cardiomyocytes using the hanging drop method. The colonies of beating EBs were counted from days 8 and 10. As shown in Figure 3A, the incidence of beating EBs in *HIF2α*-transduced cells was significantly higher than those transduced with the empty-vector construct (Figure 3A), suggesting exogenous *HIF2α* could increase the beating frequency of EBs from ESCs.

To confirm *HIF2α* treatment could promote cardiomyocyte differentiation of mouse ESCs, we detected the expressions of cardiac specific transcription factors in EBs at day 9 and found overexpression of *HIF2α* significantly increased the expression of genetic markers of cardiac progenitors; i.e., *Gata4*, *Tbx5*, *Mesp1*, *Nkx2.5* and *Mef2C* (Figure 3B).

To verify whether a full program of cardiomyogenesis occurred in *HIF2α*-ESC-derived EBs, a quantitative analysis of structural cardiomyocyte markers (i.e., *α-MHC* and *cTnT*) was performed in EBs at day 15. Higher expression of *α-MHC* and *cTnT* was observed in *HIF2α*-EBs compared with empty vector-EBs (Figure 3C). Flow cytometry (FACS) analysis was used to confirmed the cardiomyogenesis-promoting effect of HIF2α by detecting the expression of cTnT and α-SMA at day 15 (Figure 3D). Collectively, these results suggested that HIF2α is involved in cardiomyogenesis and regulates cardiac-specific transcription factors.

### HIF2α knockdown suppresses key cardiac gene expression

**Figure 4:**
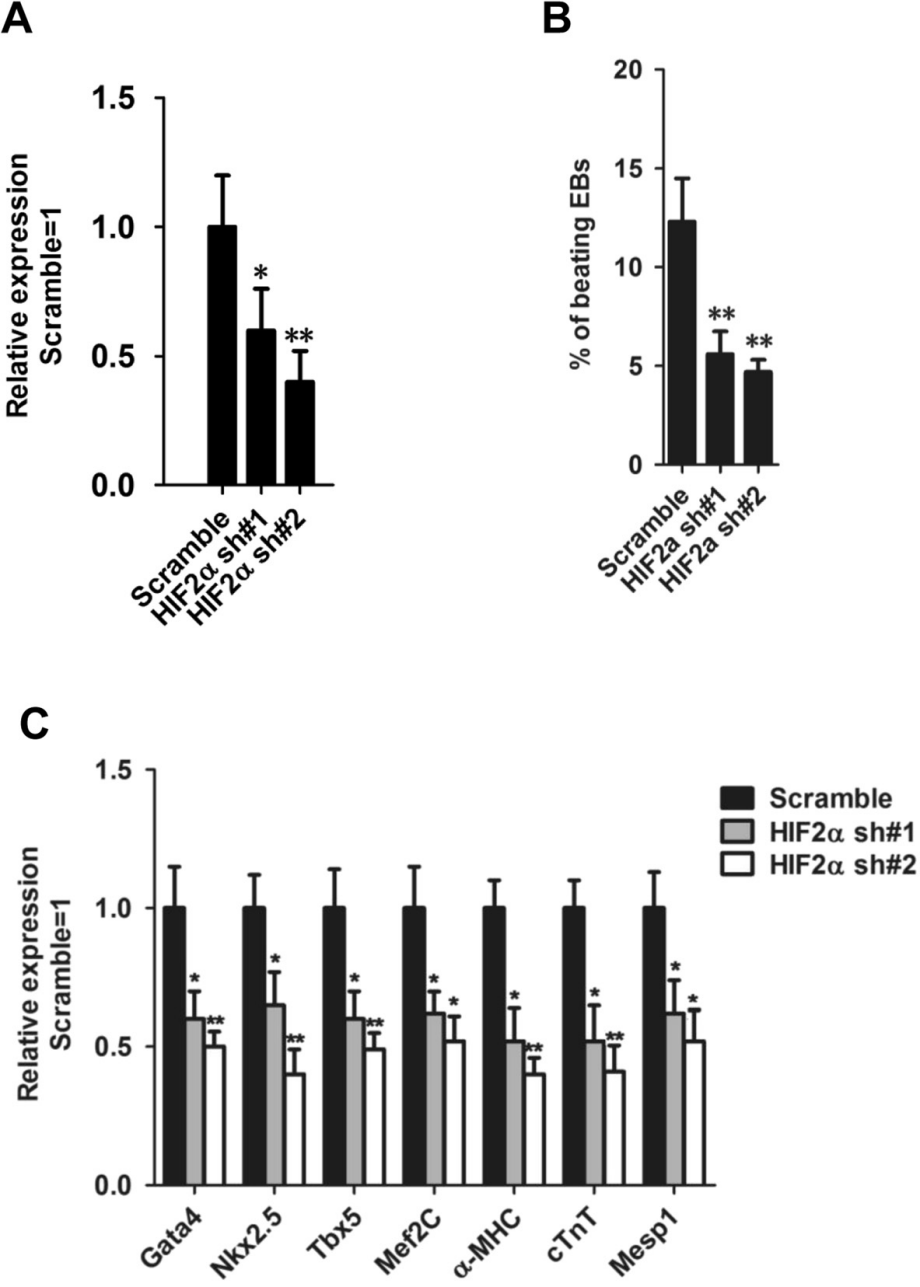
**Knockdown of HIF2α impairs the ability of ESCs for cardiac differentiation. (A)** qRT-PCR analysis of *HIF2α* mRNA levels in mouse ESCs stably transfected with *HIF2α* shRNA. The transcript level was normalized against scramble shRNA controls. Data represent the mean ± s.d. of three biological replicates. *p < 0.05, **p < 0.01 vs Scramble. **(B)** The percentages of beating EBs derived from scramble shRNA or sh*HIF2α* ESCs. Data represent the mean ± s.d. of three biological replicates. **p < 0.01 vs Scramble. **(C)** qRT-PCR analysis of cardiomyocyte-associated gene (*Gata4*, *Nkx2.5*, *Tbx5*, *Mesp1, Mef2C, α-MHC* and *cTnT*) expression in sh*HIF2α* ESC-derived EBs. Transcript levels were normalized against scramble shRNA controls. Data represent the mean ± s.d. of three biological replicates. *p < 0.05, **p < 0.01 vs Scramble.

To test whether *HIF2α* knockdown has an effect on cardiac differentiation of ESCs, we infected 46C ESCs with lentiviruses encoding two short-hairpin RNAs (shRNAs) specific for HIF2α mRNA (sh*HIF2α*). Stable knockdown of *HIF2α* transcript levels was observed following drug selection (Figure 4A). The percentage of beating EBs in *HIF2α* knockdown cells was lower than scrambled shRNA control cells (Figure 4B). sh*HIF2α* EBs showed decreased expression of cardiac markers (Figure 4C), indicating that endogenous HIF2α is important for the formation of cardiac lineages in ESCs.

### HIF2α is able to partly mimic the cardiomyogenesis-promoting effect of hypoxia

**Figure 5:**
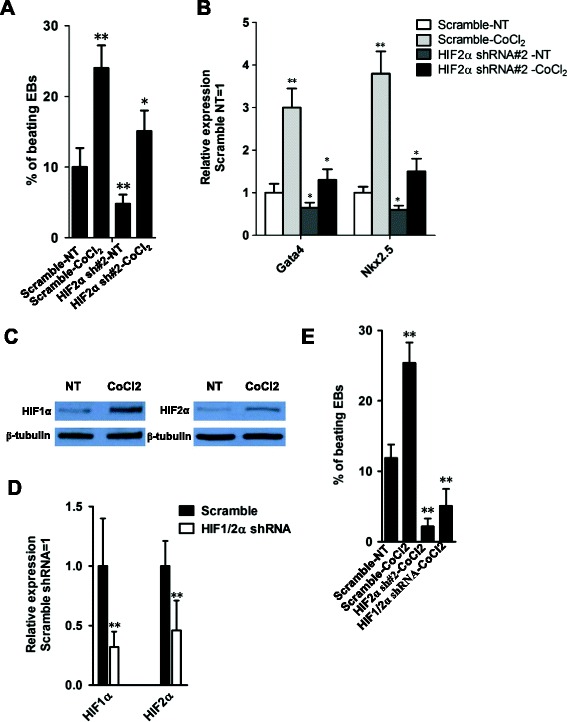
**HIF2α mediated the function of hypoxia in promoting cardiac differentiation. (A)** The percentages of beating EBs derived from scramble shRNA or sh*HIF2α* ESCs in the presence of absence of 100 μm CoCl2*.* Data represent the mean ± s.d. of three biological replicates*.* *p < 0.05, **p < 0.01 vs Scramble-NT*.* NT: No Treatment. **(B)** qRT-PCR analysis of *Gata4* and *Nkx2.5* mRNA levels in scramble shRNA or sh*HIF2α* EBs treated with or without CoCl2. Data represent the mean ± s.d. of three biological replicates. *p < 0.05, **p < 0.01 vs Scramble-NT. NT: No Treatment. **(C)** Western blot analysis of the protein levels of HIF1α or HIF2α in 46C mouse ESCs treated with or without 100 μM CoCl2 for 10 h. **(D)** qRT-PCR analysis of *HIF1α* or *HIF2α* mRNA levels in *HIF1α* and *HIF2α* double knockdown ESCs. Data represent the mean ± s.d. of three biological replicates. **p < 0.01 vs Scramble. **(E)** The beating frequency of EBs was analyzed among the indicated groups at day 15. Data represent the mean ± s.d. of three biological replicates. **p < 0.01 vs Scramble-NT.

Because hypoxia is able to induce the stabilized HIF2α protein [14], we next aimed to examine whether HIF2α is involved in the cardiac differentiation-promoting effect of hypoxia in ESCs. CoCl_2_ is known to elicit hypoxia-like responses [23], thereby we applied CoCl_2_ to chemically mimic the hypoxia-like environment during cardiac differentiation. As shown in Figure 5, 100 μM CoCl_2_ could significantly enhance the rate of beating EBs and the expression level of cardiac marker genes (Figures 5A and B), while knockdown of HIF2α impaired, albeit not totally, the promoting-effect of CoCl_2_ on cardiac differentiation (Figures 5A and B), indicating *HIF2α* partly mediated the potential cardiogenic effects of hypoxia on cultured ESCs, similar to *HIF1α* [16].

Recent studies showed areas of induction of HIF2α to a large extent overlapped with those for HIF1α in response to hypoxia and both proteins were expressed in the heart [18]. Moreover, a marked and persistent nuclear accumulation of both subunits has been observed in cardiomyocytes under systemic and regional hypoxia [24]. In addition, the hypoxic induction of *HIF1α* and *HIF2α* in isolated cardiac microvascular endothelial cells was very similar [25]. The above results indicated *HIF1α* and *HIF2α* may be functionally redundant. Consistently, we observed that 100 μM CoCl2 was able to increase the protein levels of HIF1α and HIF2α (Figure 5C). While double knockdown of *HIF1α* and *HIF2α* significantly impaired the promoting-effect of CoCl_2_ on cardiac differentiation (Figures 5E and D). However, it is worthy to note that both factors were not totally compensatory during development. For example, HIF2α protein was stabilized in type II pneumocytes and pulmonary endothelial cells in response to hypoxia, while HIF1α was not detectable [26,27]. In addition, analysis of HIF-α-staining patterns in ischemic myocardium showed that both micro- and macrovascular endothelial cells more frequently expressed HIF2α than HIF1α, and a progressive increase of HIF2α but not HIF1α occurred in myocardial tissue remote from the infarcted areas [24]. Furthermore, in the process of reprogramming, continuous and prolonged overexpression of *HIF1α* is beneficial for inducible pluripotent cell formation, while HIF2α has a stage-specific function in the process, which is essential in early, but not late, reprogramming [28]. It is of great interest to undertake more experiments to identify the specific functional redundancy between *HIF1α* and *HIF2α* during cardiac development.

### HIF2α promotes cardiac differentiation of ESCs via β-catenin activation

**Figure 6:**
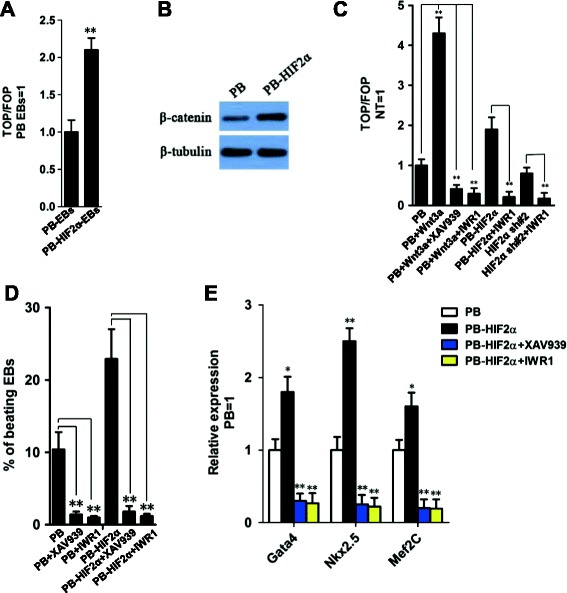
**HIF2α-mediated ESC cardiac differentiation is β-catenin-dependent. (A)** TOP Flash assay in PB- or PB-HIF2α- EBs at day 4. Data represent the mean ± s.d. of three biological replicates. **p < 0.01 vs PB-EBs. **(B)** Western blot analysis of β-catenin at day 4 PB- or PB-*HIF2α*- EBs. **(C)** TOP Flash assay of ESCs treated with the indicated conditions for 12 h. Data represent the mean ± s.d. of three biological replicates. **p < 0.01. NT: No treatment. **(D)** The percentages of beating EBs derived from PB- or PB-*HIF2α*- ESCs in the presence or absence of 2 μm XAV939 or 2 μm IWR1. Data represent the mean ± s.d. of three biological replicates. **p < 0.01. **(E)** qRT-PCR analysis of *Gata4*, *Nkx2.5* and *Mef2C* in PB- or PB-*HIF2α*- EBs in the presence or absence of 2 μm XAV939 or 2 μm IWR1. Data represent the mean ± s.d. of three biological replicates. **p < 0.01 vs PB.

A previous study reported that activation of the Wnt/β-catenin signaling pathway at an early stage of ESC differentiation will stimulate mesoderm induction and increase cardiac differentiation [8]. In addition, hypoxia has been shown to be involved in activation of the Wnt/β-catenin signaling pathway [29,30]. To determine whether this pathway is modulated by HIF2α, we transiently transfected empty vector- and *HIF2α* transgene-overexpressed 46C ESCs with a luciferase-based TOP-Flash (TCF optimal promoter) Wnt reporter plasmid, and compared reporter activity in EBs at day 4. HIF2α significantly enhanced reporter activity (2-fold; Figure 6A). Accordingly, HIF2α increased the protein level of cytosol β-catenin at day 4 (Figure 6B), suggesting that the elevated β-catenin protein may contribute to the cardiomyogenesis-promoting effect of HIF2α in ESCs.

To verify the impact of Wnt/β-catenin signaling on HIF2α induced cardiac differentiation, two β-catenin specific inhibitors; i.e., XAV939 and IWR1 [31], were used to inhibit the activity of β-catenin (Figure 6C). Inhibition of β-catenin significantly decreased the activity of Wnt/β-catenin signaling in *HIF2α* overexpressed and knockdown cells (Figure 6C). Then, the PB- and PB-*HIF2α*-transfected ESCs were incubated with XAV939 and IWR1 during the process of differentiation, and cardiac differentiation was assessed by calculating the percentage of beating EBs and detecting the cardiac cell markers *Gata4*, *Nkx2.5* and *Mef2c* at day 10. Compared with the no treatment (NT) group, both inhibitors significantly reduced the number of beating EBs and the expression of cardiac transcription factors (Figures 6D and E). These results suggested that *HIF2α*-mediated cardiogenesis is β-catenin-dependent in ESCs.

Indeed, the function of hypoxia is associated with Wnt/β-catenin signaling in many types of cells. For example, HIF1α promoted cellular adaptation to hypoxia by interacting with β-catenin [32]. HIF2α accelerated cell cycle progression and proliferation of renal cell carcinoma cells via binding β-catenin and enhancing its transcriptional activity [33]. Further study found that HIF2α could localize on the enhancer of *Wnt10b* as well as upstream of the *Wnt1* gene [34]. In cardiomyocyte development, early hypoxia lead to activation of *HIF1α* with potential subsequent effects on Wnt gene expression [29]. In our study, we also found that β-catenin is required for HIF2α to promote cardiac formation. However, it is unclear whether HIF2α has the same effect on β-catenin as HIF1α and how the latter affects β-catenin. Meanwhile, it is noteworthy that there was no significant difference seen in Luciferase activity between PB and *HIF2α* sh#2 cells unlike that of PB and PB-*HIF2α* groups (Figure 6C), implying other signaling pathways may be responsible for the cardiomyogenesis–suppressing effect of *HIF2α* knockdown. These detailed mechanisms need to be clarified further.

## Conclusions

The present results provide valuable insight on the function of HIF2α in ESC-derived cardiomyocytes. The HIF2α-induced promoting effect on β-catenin signaling may be involved in this process. Our results will be particularly helpful in attempts to achieve effective cardiac differentiation from ESCs and will provide useful information for the clinical application of ESCs.

## Additional file

Additional file 1: Table S1. Primers used for real-time PCR.

## Abbreviations

ESCs: Embryonic stem cells
HIF: Hypoxia-inducible factor
shRNA: short-hairpin RNA
EBs: Embryoid bodies
NT: No treatment
