# Supplementary material for: HIF2α induces cardiomyogenesis via Wnt/β-catenin signaling in mouse embryonic stem cells
Source: J Transl Med. 2015 Mar 14;13:88. doi: 10.1186/s12967-015-0447-7 (PMC4399227; doi:10.1186/s12967-015-0447-7)
Supplement: Additional file 1: Table S1. — Primers used for real-time PCR. [file 12967_2015_447_MOESM1_ESM.pdf]

Table S1. Primers used for real-time PCR

Gene	Forward Primer(5'→3')	Reverse Primer(5'→3')
Gapdh	TGTGAGGGAGATGCTCAGTG	TGTTCCTACCCCCAATGTGT
Oct4	GAAGCAGAAGAGGATCACCTTG	TTCTTAAGGCTGAGCTGCAAG
Sox2	ATGGGCTCTGTGGTCAAGTC	CCCTCCCAATTCCCTTGTAT
Nanog	TCCAGAAGAGGGCGTCAGAT	CAAATCCCAGCAACCACATG
HIF2α	CTAAGTGGCCTGTGGGTGAT	GTGTCTTGGAAGGCTTGCTC
Gata4	TCTCCCAGGAACATCAAAACC	GTGTGAAGGGGTGAAAAGG
Tbx5	GGAAAGATGAGGAATGTTCCAG	GTGTTACAGCTGATGTCCTCCA
Nkx2.5	GCTACAAGTGCAAGCGACAG	GGGTAGGCGTTGTAGCCATA
Mef2C	GCCCTGAGTCTGAGGACAAG	ATCAGACCGCCTGTGTTACC
Mesp1	CCTTCGGAGGGAGTAGATCC	AAAGCTTGTGCCTGCTTCAT
cTnT	CCTGCAGGAAAAGTTCAAGC	TTCCCACGAGTTTTGGAGAC
α-MHC	GATGCCCAGATGGCTGACTT	GGTCAGCATGGCCATGTCCT
